# The use of non-contrast-enhanced MRI to evaluate serial changes in endoleaks after aortic stenting: a case report

**DOI:** 10.1186/s12880-019-0379-4

**Published:** 2019-10-22

**Authors:** Yu-Li Lee, Yao-Kuang Huang, Li-Sheng Hsu, Pang-Yen Chen, Chien-Wei Chen

**Affiliations:** 1 Department of Diagnostic Radiology, Chang Gung Memorial Hospital Chiayi Branch, Chiayi, Taiwan; 2 Chang Gung University College of Medicine, Taoyuan, Taiwan; 3 Division of Thoracic and Cardiovascular Surgery, Wound Center and Plastic Surgery, Chang Gung Memorial Hospital Chiayi Branch, Chiayi, Taiwan; 4 Department of Biomedical Engineering, National Cheng Kung University, Tainan, Taiwan; 5 Department of Emergency Medicine, Mackay Memorial Hospital, Taipei, Taiwan; 6 Institute of Environmental and Occupational Health Sciences, National Yang-Ming University, Taipei, Taiwan; 7 Institute of Medicine, Chung Shan Medical University, Taichung, Taiwan; 8 Chang Gung University College of Medicine, Institute of Medicine, Chung Shan Medical University, No.6, Sec. W., Jiapu Rd, Puzi City, Chiayi County Taiwan

**Keywords:** Non-contrast-enhanced MRI, Aortic dissection, Endoleak

## Abstract

**Background:**

Aortic dissection is a life-threatening syndrome that sometimes requires emergency intervention, and endovascular aortic aneurysm repair (EVAR) is a treatment option. Long-term image follow-up is also required for patients after EVAR due to possible complications.

**Case presentation:**

We present the case of a 73-year-old male with underlying chronic renal disease diagnosed with a type A aortic dissection who underwent EVAR. Four-dimensional (three spatial dimensions combined with time) phase-contrast magnetic resonance imaging (4D PC-MRI) was performed during regular follow-up in preference to contrast-enhanced computed tomography or simple MRI while taking his poor renal function into consideration.

**Conclusions:**

We considered this preferable given his issues with renal function.

## Background

Aortic dissection is an acute syndrome in which blood enters the medial layer of the aortic wall; it is a common cause of sudden death [1]. The management options include open surgery or endovascular aortic aneurysm repair (EVAR) [2]. Patients with EVAR require long-term imaging, which is critical to detect possible complications (such as an aneurysmal expansion, rupture, or endoleakage) in a timely manner [3]. Endoleakage refers to blood flow within an aneurysmal sac after EVAR, and can be divided into five categories based on the flow characteristics [4]. It is important to define the source of blood flow into the aneurysmal sac, as it determines the type of endoleak in play, in turn informing the treatment strategy [5]. Thus, selection of an appropriate and sensitive imaging technique is essential [6].

## Case presentation

**Fig. 1:**
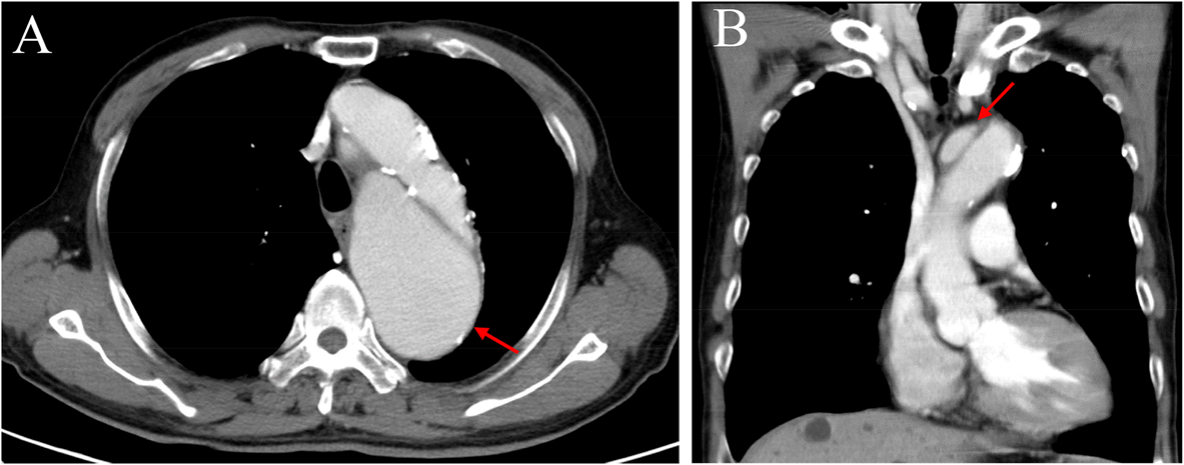
Axial (**a**) and coronal (**b**) view in contrast-enhanced computed tomography revealed dissecting flap involving the ascending and descending aorta (red arrow), and diagnosed with type A aortic dissection

**Fig. 2:**
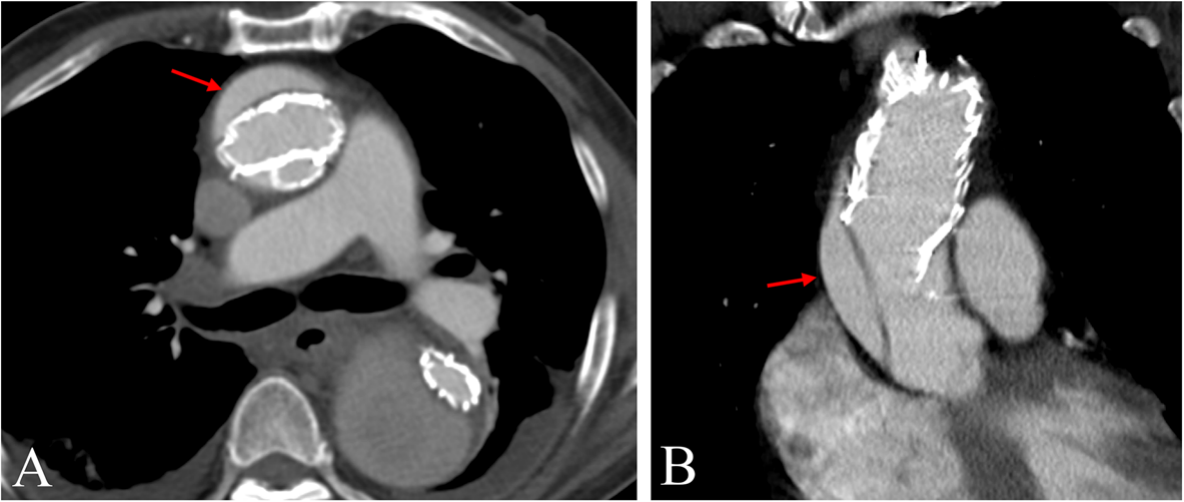
Follow-up enhancing chest CTA showed the migrating and new arising aortic dissection (red arrow) near proximal ascending aorta in axial (**a**) and coronal (**b**) view

**Fig. 3:**
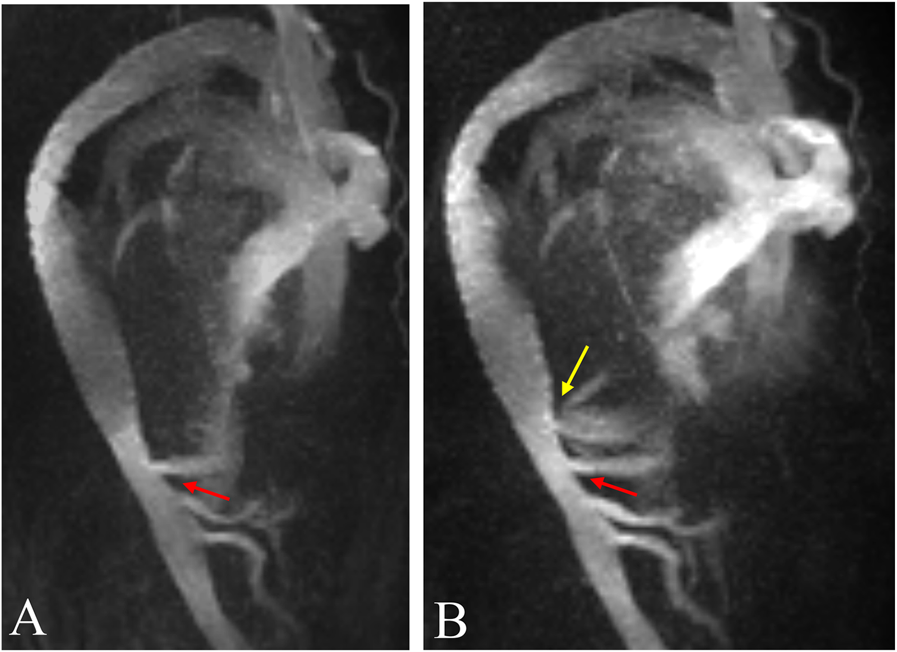
Serial 4D (three-dimensional with time dynamic) phase-contrast MRA showed type IB endoleak (red arrow) in August 2017 (**a**) changed to type IB (red arrow)-plus-type III (yellow arrow) endoleaks in July 2018 (**b**)

We report the case of a 73-year-old male with a history of medical and surgical interventions to treat aortic dissection. In January 2015, he complained of chest pain for months and rapidly progressed recently, and then was diagnosed with a type A aortic dissection with the help of contrast-enhanced computed tomography (CT) (Fig. 1). In the same month, he underwent endovascular exclusion surgery to dissect the aneurysm from the ascending to the descending aorta, and the Chimney procedure was performed to treat the innominate artery by using of the covering stents into the aortic branches instead of deployment of an endograft, which could ensure the perfusion. One week later during the same hospitalization, CT angiography (CTA) revealed a new dissecting aneurysm of the ascending aorta (Fig. 2); he underwent repeat surgery featuring arch replacement with innominate artery proximalization, and left carotid artery/left subclavian artery ligation. After discharge with stable condition, he was under regular follow up every 6 months in the first years and then annually. However, his chronic kidney disease (CKD) progressed from stage three to stage five during the follow-up period. Contrast-enhanced CTA was thus inappropriate and enhanced magnetic resonance angiography (MRA) also should be considered carefully for him. Therefore, 4D (three spatial dimensions combined with time) phase-contrast MRI (4D PC-MRI) was scheduled to avoid compromising renal function. In August 2017, follow-up 4D PC-MRI revealed a type IB endoleak, the patient decided to keep observation and regular follow up, but subsequent images in July 2018 showed the endoleaks progressed to a type IB-plus-type III (Fig. 3). We thus performed coil embolization of the aneurysmal sac.

MR images were acquired and analyzed using a 1.5-T platform (Ingenia Rev. R5 V30-rev.02; Philips, Best, the Netherlands). The patient underwent imaging in the supine position, with electrocardiographic gating. The 4D PC-MRI parameters were: 3D turbo field echo mode; shortest possible repetition time; shortest possible echo time; flip angle 5°; voxel size 2.22 × 2.27 × 3.03 mm; phase contrast velocity 120 cm/s; and scan duration 7.18 min. Imaging included the aortic arch and descending aorta. All images were acquired in the absence of gadolinium contrast medium.

## Discussion and conclusion

EVAR is used to repair aortic dissections, but commonly result in endoleaks as postoperative complications [1]. Endoleaks are classified into five types based on the direction, location, and source of blood flow; the different types require different treatments [7]. Careful screening for endoleaks after EVAR is critical in patient care.

Several imaging modalities are available. Contrast-enhanced CTA is commonly used, as it is rapid and highly sensitive [6], and multi-phase CTA can distinguish the various types of endoleaks [8]. However, unlike ultrasonography (US), CT provides no information on flow direction and rate. As some studies have suggested that arterial endoleaks may increase the risk of aneurysmal expansion and rupture, information on flow velocity and direction is important. Color Doppler US identifies the flow direction and is usually employed for rapid detection of aneurysms in emergency departments. Also, US reveals real-time dynamic changes in endoleaks or aneurysms, and is both low-cost and radiation-free. However, the data quality of US varies because of differences in operator skills and patient status. In particular, in patients with intestinal gas or those who are obese, US may not reliably diagnose aortic disease. MRA can be used to detect endoleaks after EVAR; some studies have found that the sensitivity of MRA is at least as good as that of CTA for detecting aneurysms and endoleaks [9]. However, possible nephrogenic toxicity must be considered when weighing contrast-enhanced CTA and the contrast use in MRA for patients with progressed CKD should also be avoided [10].

**Fig. 4:**
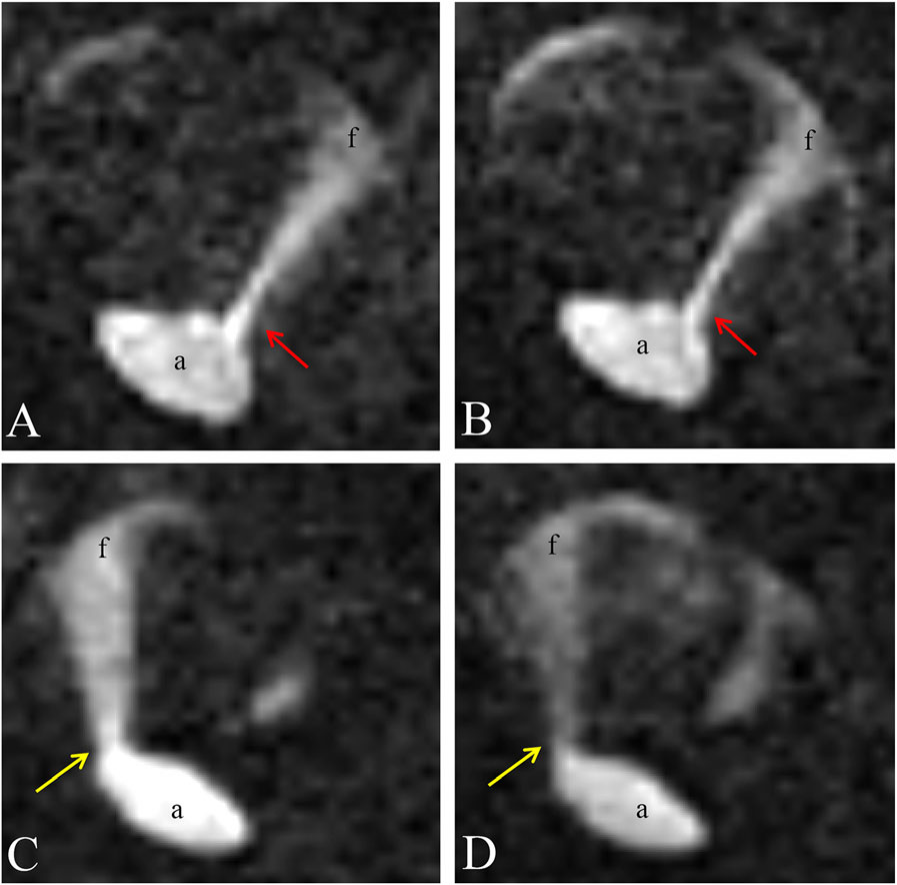
4D (three-dimensional with time dynamic) phase-contrast MRI axial view provides the information of precise location and dynamic blood flow change into the false lumens. In the follow up 4D-PC MRI, **a**, **b** revealed the type Ib endoleaks (red arrow) and **c**, **d** showed another new arising type III endoleaks (yellow arrow). (a: aorta / f: false lumens)

Our patient underwent EVAR and was followed-up every 6 months for 1 year and then annually. However, his CKD progressed to stage five from stage three during follow-up, and thus we considered contrast medium injection inappropriate. We used 4D PC-MRI as an alternative; the aortic stents were fabricated from nitinol. During follow-up, 4D PC-MRI showed that the endoleak changed from type IB to a combination of type IB/type III. Since 4D PC-MRI revealed the dynamics of flow between the aortic stent and false lumens (Fig. 4), we were able to define the various endoleaks in play. 4D PC-MRI revealed the precise locations of, and blood pooling within, false lumens; we were able to compare endoleak status before and after treatment. Besides, the 4D PC-MRI can also provide flow information and luminal anatomy with excellent background signal suppression in one single scan. In conclusion, we thought the 4D PC-MRI can be used to detect endoleaks after EVAR in patients with CKD, and is a valuable clinical alternative to contrast-enhanced CTA or MRA.

## Data Availability

All data generated or analyzed during this study are included in this published article. (MRI sequences included in the section of case presentation).

## Abbreviations

4D PC-MRI: Four-dimensional (three spatial dimensions combined with time) phase-contrast magnetic resonance imaging
CKD: Chronic kidney disease
CT: Computed tomography
CTA: CT angiography
EVAR: Endovascular aortic aneurysm repair
MRA: Magnetic resonance angiography
US: Ultrasonography
